# Off-label magnetic resonance imaging of an InterStim II sacral nerve stimulator device

**DOI:** 10.1259/bjrcr.20170064

**Published:** 2017-10-13

**Authors:** Andrew Fry, Nikhil Kotnis, Russel Edward, Peter J Wright

**Affiliations:** Department of Medical Imaging and Medical Physics, Sheffield Teaching Hospitals NHS Foundation Trust, Sheffield, UK

## Abstract

The InterStim II (Medtronic, Inc., Minneapolis, MN) sacral nerve stimulator has been approved for MRI scanning of the head only. All other body areas are contraindicated by the manufacturer. This report presents the successful MRI examination of the left hand in a patient with an InterStim II device. Following an assessment of the risks and benefits of proceeding with the scan it was shown that there were minimal additional risks, which could be easily managed with appropriate patient positioning, coil selection and other established techniques. Informed consent was obtained and the scan completed without incident. Following the scan the patient reported full functioning of the device. MRI of the hand is feasible in patients with InterStim II implants using transmit/receive coils with appropriate risk controls in place. Further study of the safety of MRI of other body regions in InterStim II patients is appropriate.

## Introduction

The InterStim II sacral nerve stimulator (SNS) (Medtronic Inc., Minneapolis, MN) is indicated for urinary or bowel control for treatment of urinary retention, overactive bladder symptoms and chronic faecal incontinence in patients where conservative treatment has failed.^1^ It is not unusual for these patients to have comorbidities that require the use of MRI, with patient numbers only likely to increase as demand for MRI grows year on year. In England alone the number of MRIs performed has grown 276% during the 10 years from 2006 to 2016 and is now the equivalent of one MRI scan per 21 of the population per year.^2^

Currently, the manufacturer recommends that MRI scanning of the head only may be performed on InterStim patients, under very specific conditions (Table 1). This is due to concerns about dislodgement of the device, unintended stimulations and, especially, heating of the leads and device.^3^ There are similar concerns about other active implanted devices, but an increasing number of studies have shown that MRI can be performed with no adverse effects in patients with pacemakers, spinal cord stimulators, deep brain stimulators, vagus nerve stimulators and cochlear implants.^4,5 ^

**Table 1.** Manufacturer's recommended scanning conditions and proposed scanning conditions

Manufacturer’s guidelines^3^	Proposed scan conditions
1.5 T closed bore	1.5 T closed bore
RF transmit/receive head coil only (no RF transmit body coil)	RF transmit/receive **knee** coil (no RF transmit body coil). Patient in prone “superman” position, hand placed in knee coil
Max. spatial gradient 19 T m^–1^	Max. spatial gradient 19 T m^–1^
Max. gradient slew rate 200 T m^–1^ s^–1^	Max. gradient slew rate 200 T m^–1^ s^–1^
Normal operating mode. Whole body SAR < 2 W kg^–1^	Normal operating mode. Whole body SAR < 2 W kg^–1^
Do not sedate patient if possible	Patient not sedated
Model 3058—turn the neurostimulator off	Model 3058—turn the neurostimulator off

Max, maximum; RF, radio frequency; SAR, specific absorption rate.

Previous studies have demonstrated no serious adverse outcomes after patients with the Medtronic ITRELL and Medtronic InterStim (model 3023) SNS had MRI of the head, spine, pelvis and foot.^6–8^ This report describes the successful MRI examination of the hand of a patient with an InterStim II (model 3058) device. To the best of our knowledge there have been no reported off-label MRI scans of this device.

## Presentation

A 53-year-old female was referred for MRI examination of the left hand for further characterization of a mass following an ultrasound examination. On ultrasound the appearance of the mass was indeterminate. The patient had the SNS system (InterStim II 3058 generator with 3093 lead electrode) implanted 7 years previously (2009) and the battery had been changed 3 months prior to the MRI. While this system is classed as MR Conditional for head scans (using a transmit/receive (Tx/Rx) head coil), other body areas are not recommended by the manufacturer.

## Investigation

The Medicines and Healthcare products Regulatory Agency (MHRA) have produced guidance documents for off-label use of a medical device and for scanning patients with implants where MRI may be contraindicated.^9,10^ Following the MHRA guidelines a risk assessment was conducted by an MR Clinical Scientist. Risk assessment determined the presence and severity of any additional risks and was used to evaluate existing and additional control measures that might be implemented to mitigate the risks where possible (Table 2). A comparison of the manufacturer’s recommended scan conditions and the proposed scan conditions is shown in Table 1.

**Table 2.** Risk assessment for off-label scanning of the hand of a patient with an InterStim II device

Hazard	Cause	Comparative risk with reference to head scan	Additional precautions	Controls in place
Mechanical stress/ displacement	Static magnetic field; incomplete fibrosis	Comparable	None: risk already controlled	>6 weeks since implantation
Induced stimulation	Gradient fields; RF absorption; Ohmic heating	Reduced: transmit/receive coil is further from device	None: risk already controlled	Tx/Rx coil. No part of device within coil. Device turned off
Discomfort	Gradient fields	Comparable or slightly increased: Patient positioned prone so body weight may not prevent device movement as when supine	None: risk already controlled	Patient unsedated and given call buzzer. Communicate with patient between sequences. Some vibration/tugging of device possible; stop scan if uncomfortable
Heating and/or burns	RF absorption; Ohmic heating	Reduced: transmit/receive coil is further from device	None: risk already controlled	Patient unsedated and given call buzzer. Communicate between sequences. Stop scan if heating felt
Image artefacts	Magnetic inhomogeneity; patient movement	Comparable: transmit/receive coil is further from device; however, patient positioning harder to tolerate	Foam padding around hand to limit movement	Device not within field of view
Device reset	Gradient field; pulsed RF field	Comparable	None: risk already controlled	Device can be reprogrammed with clinician programmer. Nurse specialist to attend pre- and post-MRI to confirm device working

RF, radio frequency.

At this institution a patient with this clinical presentation and no special considerations would be scanned at 3.0 T with a dedicated hand and wrist coil. The opinion of an experienced musculoskeletal consultant radiologist was that a scan at 1.5 T with a larger coil, while providing lesser image quality, would still be diagnostic.

The heating risk increases with increased radiofrequency (RF) power deposition and RF emission duration. This is considered in the specific absorption rate (SAR) calculations performed by the scanner for each sequence. By imposing SAR limits the risk of damage due to heating can be reduced.

The manufacturer’s guidelines for MRI scanning of patients with this implant stipulates that only a Tx/Rx head coil may be used. With this type of coil RF power is largely deposited in the tissue within the coil, dramatically limiting the potential for heating elsewhere.^11^ In addition, exposing the device to the RF emissions may induce unwanted stimulations. The risk assessment identified that so as not to vastly increase the risk of heating and unintended stimulation, a Tx/Rx coil must be used for the hand. The risk of heating was further mitigated by allowing rest periods after each sequence to give time for physiological thermoregulatory processes to dissipate any heat around the device.

The standard hand coil is a receive-only type and therefore a Tx/Rx knee coil was identified as the most appropriate coil. The size of the coil prevented it from being placed at the patient’s side, in the standard hand coil position. The patient was therefore positioned prone, with the arm extended above the head (the so-called “superman” position). Because of the “superman” positioning of the patient it was considered that the risk of heating and stimulation was slightly reduced compared to a head scan because the device and leads were further away from the RF transmission source (*i.e.* the coil).

There was potential for increased movement artefacts on the images due to the patient position, which can be difficult to tolerate. The patient was confident that she could tolerate the position for the duration of the scan and foam padding was used to stabilize the hand within the coil.

## Outcome

Informed consent was obtained from the patient by the MR Safety Expert. The patient’s device was turned off with the patient programmer according to the manufacturer’s instructions. The patient was positioned prone on the MR scanner table, with the left arm raised above her head in the“superman” position. The left hand was positioned inside the Tx/Rx knee coil and padded with foam to limit movement. MRI continued uneventfully using a standard hand protocol with all sequences having a whole body SAR much less than 2 W kg^–1^, as displayed on the scanner console during scanning and reported in the DICOM header information(Table 3). Communication between the MR radiographer and the patient was maintained between sequences and the patient reported no discomfort or sensations of heating. Diagnostic images were obtained and the SNS device was successfully reactivated using the patient programmer following the scan. The patient reported normal function of the system following the MRI examination.

**Table 3.** Reported whole body SAR values for each sequence used in the MRI examination from DICOM tag (0018, 1316)

Sequence	Whole body SAR [W kg^–1^]
Localizers 2D RF-spoiled Gradient Echo	0.002
T_1_ Fast Spin Echo—Coronal	0.126
T_1_ Fast Spin Echo—Axial	0.106
T_2_ Fast Spin Echo with fat saturation—Coronal	0.117
PD Fast Spin Echo with fat saturation—Sagittal	0.066
PD Fast Spin Echo with fat saturation—Axial	0.140
T_1_ 3D Ultrafast Gradient Echo—Sagittal	0.005

The MRI examination allowed further characterization of the mass compared to ultrasound. Axial *T*_1_ images appeared to show a non-fatty component to the lesion, making it possible that it was not a simple lipoma. The patient was referred to the sarcoma multidisciplinary team for urgent follow-up.

## Conclusions

This case demonstrates that MRI scanning at 1.5 T using a transmit/receive extremity coil may be carried out safely for this SNS system, following a suitable risk assessment and implementation of risk reduction measures. Institutions wishing to scan patients with SNS devices contraindicated for MRI should follow MHRA advice^9,10^ and produce a risk assessment considering local hazards and variations in addition to the risks highlighted in this report. Further study would be helpful in establishing the safety of MRI of other body regions for patients with InterStim II devices.

## Learning points

1. The MRI scanning procedure described is contraindicated under the manufacturer’s MRI guidelines; however, scanning may be performed without incident after a suitable risk/benefit analysis is performed and risk control measures are implemented.
2. In some cases manufacturers' MRI guidelines may be restrictive due to lack of testing of alternative scan techniques, rather than the presence of additional risk when using these techniques.
3. Off-label MRI scanning should be approached with caution and follow MHRA published guidelines.

## Consent

Written Informed consent to publish this case report was obtained from the patient.
